# Glycolate Oxidase Isozymes Are Coordinately Controlled by *GLO1* and *GLO4* in Rice

**DOI:** 10.1371/journal.pone.0039658

**Published:** 2012-06-26

**Authors:** Zhisheng Zhang, Yusheng Lu, Liguang Zhai, Rongshu Deng, Jun Jiang, Yong Li, Zhenghui He, Xinxiang Peng

**Affiliations:** 1 State Key Laboratory for Conservation and Utilization of Subtropical Agro-bioresources, South China Agricultural University, Guangzhou, China; 2 Laboratory of Molecular Plant Physiology, College of Life Sciences, South China Agricultural University, Guangzhou, China; 3 Department of Biology, San Francisco State University, San Francisco, California, United States of America; Friedrich-Alexander-University Erlangen-Nurenberg, Germany

## Abstract

Glycolate oxidase (GLO) is a key enzyme in photorespiratory metabolism. Four putative GLO genes were identified in the rice genome, but how each gene member contributes to GLO activities, particularly to its isozyme profile, is not well understood. In this study, we analyzed how each gene plays a role in isozyme formation and enzymatic activities in both yeast cells and rice tissues. Five GLO isozymes were detected in rice leaves. *GLO1* and *GLO4* are predominately expressed in rice leaves, while *GLO3* and *GLO5* are mainly expressed in the root. Enzymatic assays showed that all yeast-expressed GLO members except GLO5 have enzymatic activities. Further analyses suggested that GLO1, GLO3 and GLO4 interacted with each other, but no interactions were observed for GLO5. GLO1/GLO4 co-expressed in yeast exhibited the same isozyme pattern as that from rice leaves. When either *GLO1* or *GLO4* was silenced, expressions of both genes were simultaneously suppressed and most of the GLO activities were lost, and consistent with this observation, little GLO isozyme protein was detected in the silenced plants. In contrast, no observable effect was detected when *GLO3* was suppressed. Comparative analyses between the GLO isoforms expressed in yeast and the isozymes from rice leaves indicated that two of the five isozymes are homo-oligomers composed of either GLO1 or GLO4, and the other three are hetero-oligomers composed of both GLO1 and GLO4. Our current data suggest that GLO isozymes are coordinately controlled by *GLO1* and *GLO4* in rice, and the existence of GLO isozymes and GLO molecular and compositional complexities implicate potential novel roles for GLO in plants.

## Introduction

Glycolate oxidase (GLO) is a key enzyme in photorespiration and catalyzes the oxidation of glycolate to glyoxylate, with an equimolar amount of H_2_O_2_ produced [1]. Noctor et al. estimated that more than 70% of the total H_2_O_2_ production in photosynthetic leaves of C_3_ plants comes from photorespiration via GLO catalysis [2]. In addition to its known function in photorespiration, studies have suggested that GLO may also play roles in plant stress responses. It has been frequently observed that GLO activities were induced in response to various environmental stresses, including drought stress, which was observed in *Vigna*, pea and tobacco [3]–[5]. GLO has been implicated in plant resistance to pathogens [6]–[8], and more intriguingly, recent studies demonstrated that GLO is an alternative source for the production of H_2_O_2_ during both gene-for-gene and non-host resistance in *Nicotiana benthamiana* and *Arabidopsis* [9]. As described above, the recognition of the importance of GLO by researchers has been increasing, and further insights into its molecular and biochemical properties are of both scientific and practical significance.

Historically, extensive screens were conducted to identify specific inhibitors that suppress GLO activities [10]–[11] in order to analyze its functions. However, these attempts turned out to be unsuccessful. While mutants that are deficient in various photorespiratory enzymes were subsequently identified, GLO-lacking mutants never showed during this genetic screening approach [12]. Yamaguchi and Nishimura (2000) isolated several transgenic tobacco lines with *GLO* co-suppressed [13]. Subsequently, Zelitch et al. (2009) identified activator insertional maize mutants with GLO defects [14]. We were able to suppress GLO activities in rice by using an inducible antisence system [15]. Interestingly, in all these reports it was consistently observed that plants with GLO defects showed the typical “photorespiratory phenotype” That is, transgenic plants with GLO defects are lethal in air but normal under high CO_2_. This phenotype is consistent with what was observed in mutants with defects of the other photorespiratory enzymes, such as 2-phosphoglycolate phosphatase (PGP), serine:glyoxylate aminotransferase (SGAT), serine hydroxymethyitransferase (SHMT), glycine decarboxylase complex (GDC), hydroxypyruvate reductase (HPR), and glycerate kinase (GLK) [12], [16]. The observation of the photorespiratory phenotype in maize plants defective in GLO activity suggests that either the photorespiratory pathway is equally important in C_4_ plants as it is in C_3_ plants [14], or that GLO plays a second essential, yet unidentified, role in plants, which has been previously proposed by Somerville and Ogren [17].

While appreciable work has been done on both the catalytic and biochemical properties of GLO in plants, very inconsistent data have been obtained. For instance, the reported molecular weight of GLO has ranged from 88 to 700 kDa, corresponding to a subunit number from 2 to 16. Additionally, measured pIs for GLO have ranged from 7.5 to 9.6 [18]–[27]. It has been generally accepted that GLO is a homo-oligomer that exists as a single form in plants [1], but isoforms have been demonstrated in tobacco and maize plants [28]–[29]. Determining the precise nature of GLO isozymes in plants, and their detailed biological functions, are critical to understanding GLO in plants.

In this study, we detected the presence of GLO isozymes in rice leaves, and identified and characterized their corresponding *GLO* genes. A series of further analyses, such as heterologous expressions, interaction assays, isozyme pattern comparison, and specific gene silencing, have advanced our understanding of the molecular and biochemical aspects of GLO in rice.

## Results

**Figure 1:**
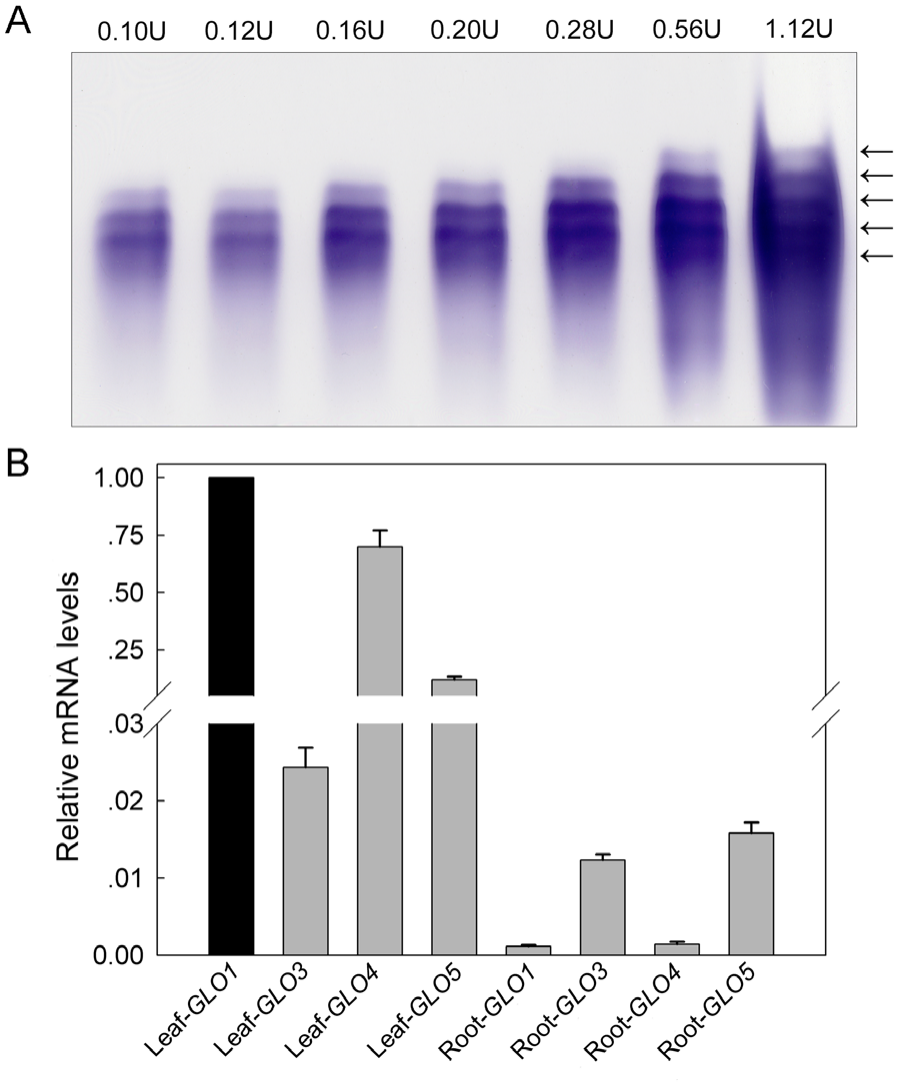
GLO isozyme patterns and the expression of various *GLO* members in rice. (**A**) Enzymatic activity staining showing patterns of GLO isozymes. GLO enzyme was extracted from rice leaves and separated by 6% CN-PAGE at a running pH of 10.2. The number above each lane indicates activity units (µmol H_2_O_2_ min^−1^ mg^−1^ protein) loaded. Arrows point to each isozyme band. This result is representative of five independent experiments. (**B**) mRNA transcript abundance of the four *GLO* genes (*GLO1, GLO3, GLO4,* and *GLO5*) in rice leaves and roots was determined by real-time quantitative RT-PCR. The second leaf from the top and 5 cm of roots were detached from plants for RNA isolation. Relative mRNA levels were graphed based on the mRNA level of Leaf-*GLO1* as 1. The data represent means ±SD of 3 independent experiments.

During our long-term study of GLO in plants, we used chromatography in an attempt to separate the GLO isozymes of rice. The goal was to isolate each isozyme so that their individual biochemical and catalytic properties could be studied. Unfortunately, likely due to the high similarity of the proteins (Table S1), such efforts turned out to be unsuccessful. Alternatively, we utilized a modified clear-native PAGE (CN-PAGE) system to examine GLO isozymes. By this approach, we successfully detected five GLO isozymes in rice leaves (Figure 1A). The three bands in the middle were the most abundant, with an order of the second > the third > the fourth. The first and fifth bands were relatively weak, and were seen only when a high amount of enzyme extract was loaded (Figure 1A).

**Figure 2:**
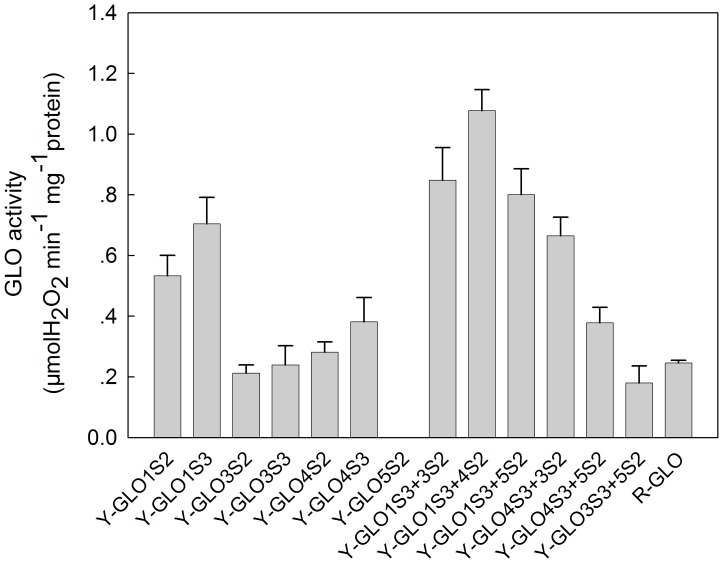
Catalytic activities of the GLO isoforms expressed in yeast. Y-GLO1S2, Y-GLO3S2, Y-GLO4S2, Y-GLO5S2 represent the crude enzyme extracted from yeast cells expressing pYES2-*GLO1*, pYES2-*GLO3*, pYES2-*GLO4*, pYES2-*GLO5*, respectively. Y-GLO1S3, Y-GLO3S3, Y-GLO4S3, represent the crude enzyme extracted from yeast cells expressing pYES3-*GLO1*, pYES3-*GLO3*, pYES3-*GLO4*, respectively. Y-GLO1S3+3S2, Y-GLO1S3+4S2, Y-GLO1S3+5S2, Y-GLO4S3+3S2, Y-GLO4S3+5S2, Y-GLO3S3+5S2, represent the crude enzyme extracted from yeast cells co-expressing pYES3-*GLO1* and pYES2-*GLO3*, pYES3-*GLO1* and pYES2-*GLO4*, pYES3-*GLO1* and pYES2-*GLO5*, pYES3-*GLO4* and pYES2-*GLO3*, pYES3-*GLO4* and pYES2-*GLO5*, pYES3-*GLO3* and pYES2-*GLO5*, respectively. R-GLO represents the crude enzyme extracted from leaves of rice. The data represent means ±SD of 3 independent experiments.

**Figure 3:**
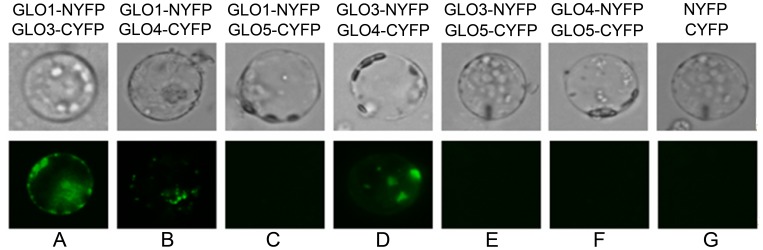
BiFC detection of interactions among the proteins encoded by the four *GLO* genes. Every two *GLO* genes were co-expressed in rice protoplasts and BiFC visualization assays were carried out to test the interaction as indicated: (A) NYFP-tagged GLO1 and CYFP-tagged GLO3; (B) NYFP-tagged GLO1 and CYFP-tagged GLO4; (C) NYFP-tagged GLO1 and CYFP-tagged GLO5; (D) NYFP-tagged GLO3 and CYFP-tagged GLO4; (E) NYFP-tagged GLO3 and CYFP-tagged GLO5; (F) NYFP-tagged GLO4 and CYFP-tagged GLO5; (G) NYFP and CYFP. This result is representative of three independent experiments.

A search of the rice genome identified six similar sequences that are annotated as putative glycolate oxidases. These sequences are located on chromosomes 3, 4, 7 and 8, respectively, and thereby designated as *GLO1* through *GLO6* according to their order on the chromosomes. Out of the six sequences, only four of them have credible ORFs, *i. e., GLO1*, *GLO3*, *GLO4* and *GLO5*, which encode proteins with 369, 367, 369 and 366 amino acids, respectively. Similarity between these proteins is appreciably high (Table S1). Transcriptional analyses showed that the mRNA levels of the four genes are much higher in leaves than in roots (Figure 1B). Relative levels of expression were *GLO1* ≈ *GLO4*>> *GLO5*>> *GLO3* in leaves, and *GLO3* ≈ *GLO5*>> *GLO1* ≈ *GLO4* in roots (Figure 1B). This pattern was not significantly changed during different growth stages (data not shown). To evaluate the contribution of the four genes to GLO activities, each individual GLO gene was heterologously expressed in yeast. Enzymatic activity assays found that, GLO1, GLO3 and GLO4 showed appreciable glycolate oxidase activities, whereas GLO5 lacked activity (Figure 2). The order of activity strength for the assay was GLO1> GLO4> GLO3. In addition, when GLO1/GLO4, GLO1/GLO3, or GLO3/GLO4 pairs were co-expressed in yeast, the activity was almost additively increased, whereas no increase occurred when GLO5 was co-expressed with any of the other three GLOs (Figure 2). Bimolecular fluorescence complementation technique (BiFC) analyses demonstrated that GLO1, GLO3 and GLO4 were able to physically interact with each other. Co-expression of GLO1 and GLO4 resulted in bright, point-like fluorescence, whereas the fluorescence of GLO3 and GLO1, GLO3 and GLO4 was more dispersed. GLO5 did not physically interact with the other isoforms in this assay (Figure 3), a result that was further confirmed by His-tag pull down assay (Table 1).

**Table 1:** Interactions of the GLOs determined by His-tag pull down assay.

Co-expressed GLOs	Activity recovery (%)
GLO1-his + GLO3	91.63%
GLO1-his + GLO4	96.79%
GLO3-his + GLO1	65.99%
GLO3-his + GLO4	35.65%
GLO4-his + GLO1	88.16%
GLO4-his + GLO3	68.47%
GLO5-his + GLO1	4.51%
GLO5-his + GLO3	5.71%
GLO5-his + GLO4	2.99%

GLO-his means the 6 amino acids on the C-terminus of GLO was mutated to a 6×his-tag. The interactions between every two GLOs are evaluated by calculating the activity recovery rate. The data are means of 3 independent experiments.

**Figure 4:**
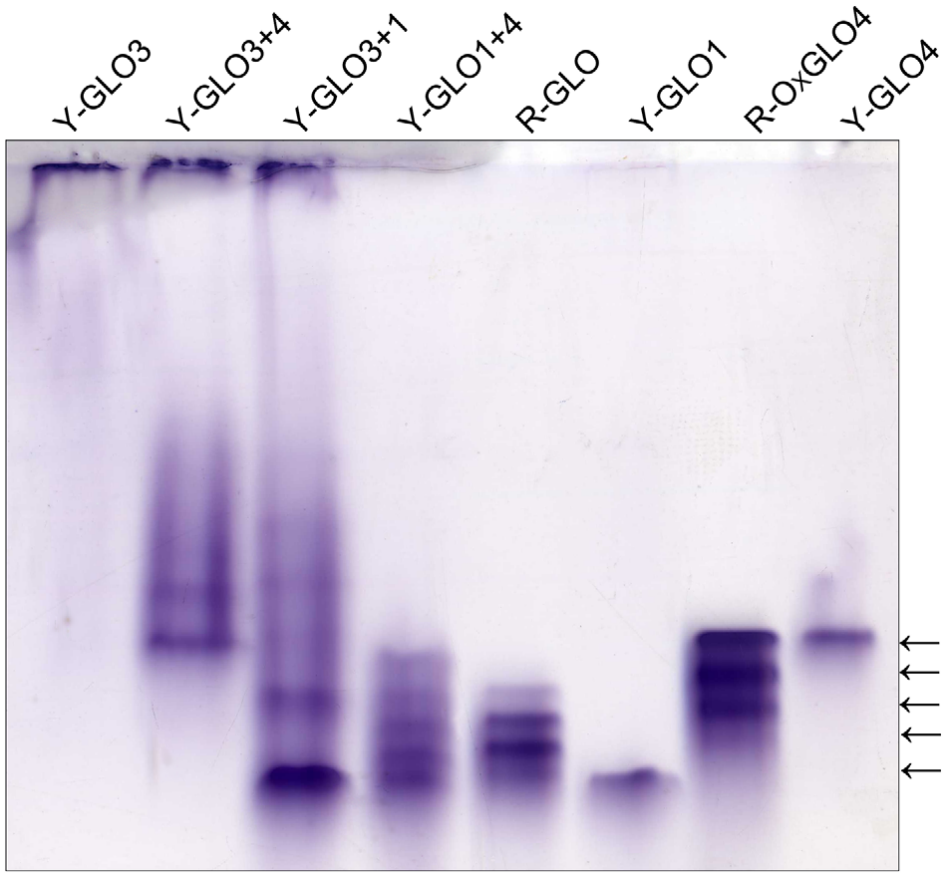
Comparison between the GLO isoforms expressed in yeast and those extracted from rice leaves. GLO isozymes were separated in 6% uniform CN-PAGE at a running pH of 10.2. Y-GLO1, Y-GLO3, Y-GLO4, represent the enzyme extracted from yeast cells expressing pYES3-*GLO1*, pYES3-*GLO3*, pYES3-*GLO4*, respectively. Y-GLO3+4, Y-GLO3+1, Y-GLO1+4, represent the enzyme extracted from yeast cells co-expressing pYES3-*GLO3* and pYES2-*GLO4*, pYES3-*GLO3* and pYES2-*GLO1*, pYES3-*GLO1* and pYES2-*GLO4*, respectively. R-GLO represents the enzyme extracted from leaves of rice, and R-OxGLO4 represents the enzyme extracted from leaves of *GLO4*-overexpressed transgenic rice. This result is representative of five independent experiments.

**Figure 5:**
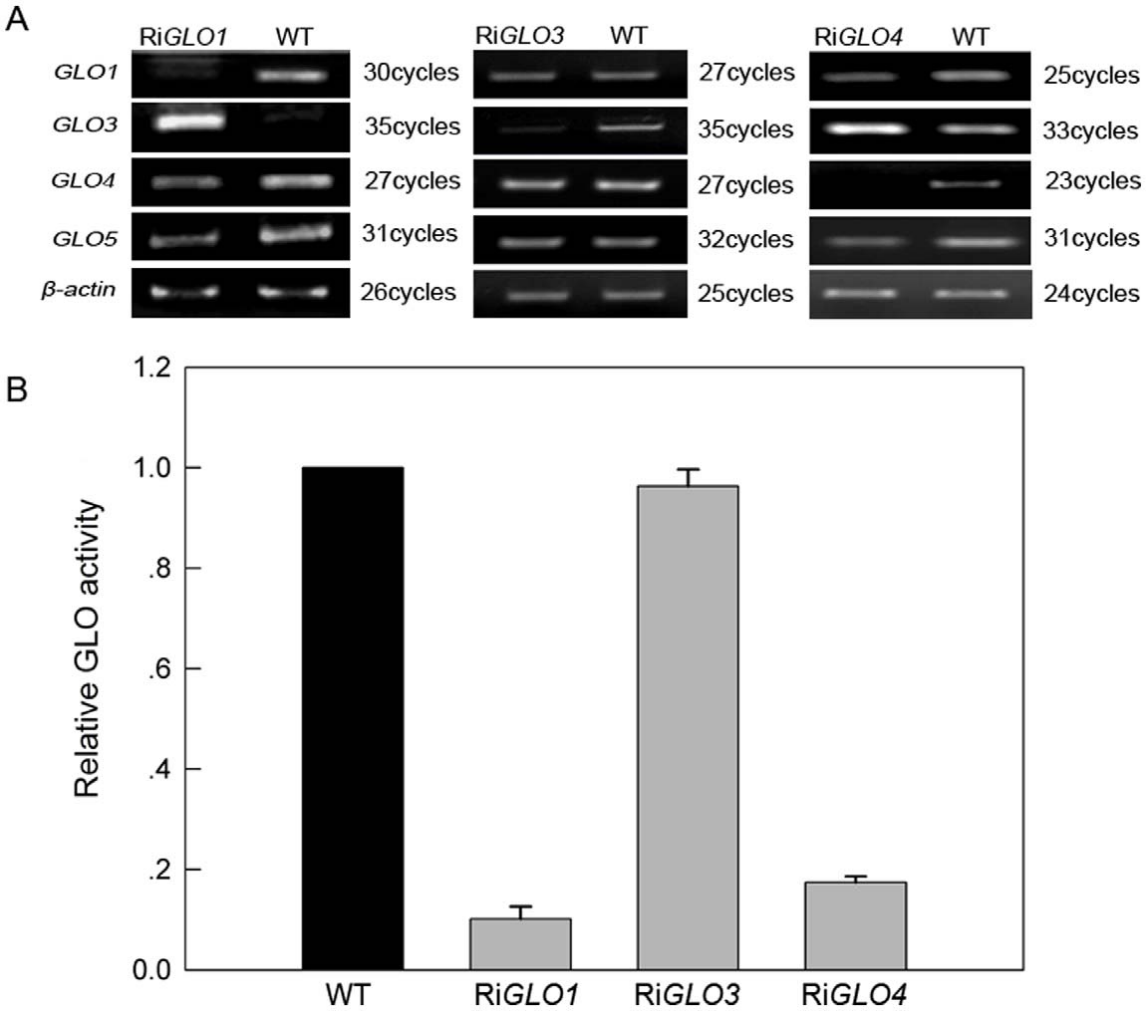
*GLO1* and *GLO4* are the major contributors to GLO activities in rice. (**A**) Semi-quantitative RT-PCR analysis of each GLO gene in the transgenic plants carrying the silencing construct. This result is representative of three independent experiments. (**B**) GLO enzyme activities in transgenic plants. Ri*GLO1*, Ri*GLO3*, Ri*GLO4* represent the specific *GLO1*, *GLO3* and *GLO4* RNA-silencing transgenic plants, respectively. The second leaf from the top was detached from plants at vegetative stage. Relative GLO activity was graphed based on the GLO activity of wild type (WT) as 1. The data represent means ±SD of 3 independent experiments.

We then compared the isozyme profiles between the isoforms expressed in yeast and the GLO from rice leaves. As shown in Figure 4, when either GLO1 or GLO4 was expressed in yeast, a single band was seen, whose position was comparable to the first and fifth bands for the rice GLO. When the genes were pair by pair co-expressed in yeast, only GLO1/GLO4 exhibited the same isozyme pattern as the GLO pattern seen in rice. When GLO4 was over-expressed in rice, the fifth band became much stronger, whose position was comparable to GLO4 expressed in yeast. GLO3 could barely move through the gel in this system, and the isozyme patterns for GLO3/GLO4 and GLO3/GLO1 were obviously different from the rice GLO pattern (Figure 4). Up to this point, our results suggest that *GLO1* and *GLO4* control GLO activities and GLO isozyme formation. To further verify this observation *in vivo*, an RNAi approach was used to specifically silence each *GLO* gene. The interfering sequences were carefully designed to guarantee the specificity of silencing (Table S2). When either *GLO1* or *GLO4* was down-regulated, expression of both genes was simultaneously suppressed, and most of the GLO enzymatic activities were lost. (Figure 5). Consistent with this observation, little GLO isozyme proteins were detected in these plants (data not shown).

**Figure 6:**
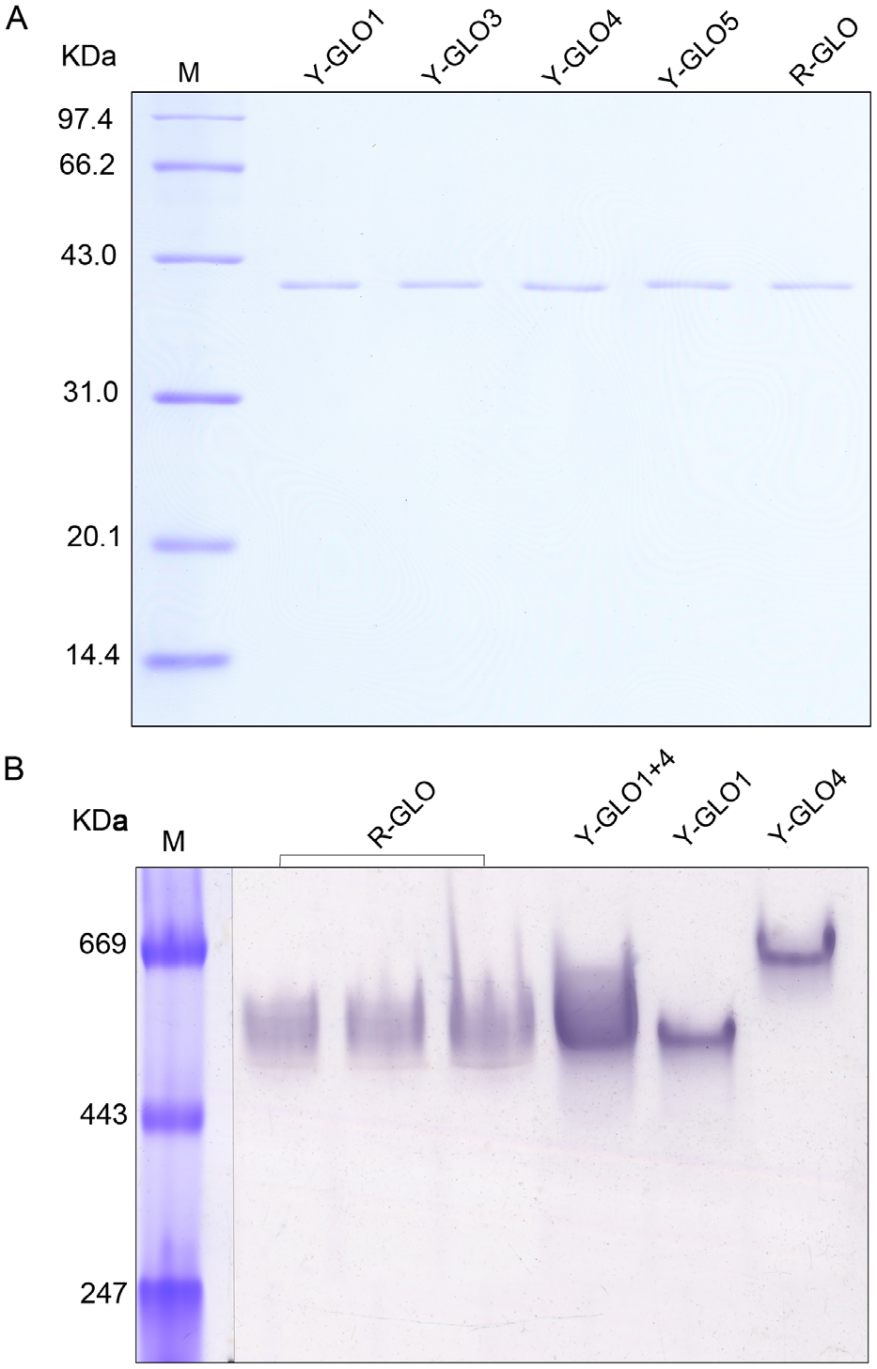
Molecular weights of the GLO isofroms extracted from yeast and rice leaves. (**A**) The molecular weights of the subunits determined by uniform SDS-PAGE (12.5%). Y-GLO represents the enzyme purified from yeast cells by immobilized metal affinity chromatography, and R-GLO represents the enzyme purified from leaves of rice. (**B**) The molecular weights of the holoenzymes determinated by gradient CN-PAGE (3–12%, running pH 8.3). Y-GLO represents the crude enzyme extracted from yeast cells, R-GLO represents the partially purified enzyme extracted from leaves of rice. This result is representative of five independent experiments.

SDS-PAGE analyses indicated that the subunit size for GLO1, GLO3, GLO4 and the GLO enzyme detected in rice leaves were all identically at 40 kDa (Figure 6A). Further gradient CN-PAGE analysis showed that the molecular weight of the rice GLO isozymes ranged from 490 kDa to 650 kDa (Figure 6B). From this we deduced that the subunit number was from 12 to 16, and that the first isozyme was a 12-mer composed uniformly of GLO1, and that the fifth isozyme was a 16-mer composed uniformly of GLO4. The other three isozymes were hetero-oligomers composed of GLO1 and GLO4. It is predicted by the ExPASy Proteomics Server that the pIs of GLO1, GLO3, GLO4, GLO5 are 8.5, 9.1, 8.5, 8.5, respectively. Since we observed that the rice GLO isozymes migrated on the CN-PAGE gel with a running pH of 8.3 (Figure 6B), the pIs for these isozymes should be lower than 8.3. Further, since the isozymes were retained on the DEAE-sepharose when the column was flushed with a pH 7.8 buffer (refer to the method for more details), their pIs should be even lower than 7.8. The exact pIs for these GLO isozymes wait to be more quantitatively determined.

**Figure 7:**
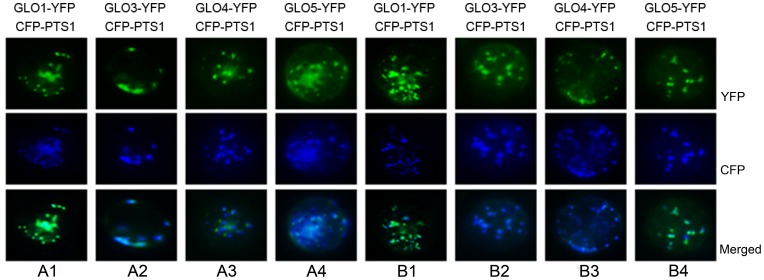
Subcellular localization of GLO isoforms. The YFP-tagged GLOs and CFP-tagged PTS1 fusion constructs were used in protoplast transient expression system to determine the subcellular localization of GLOs, the CFP-tagged PTS1 was used as the peroxisome marker. A1–A4: transfection of rice protoplasts; B1–B4: transfection of *Arabidopsis* protoplasts. This result is representative of three independent experiments.

Subcellular localization analyses reveal that each GLO isoform harbors a peroxisomal signal pepetide (PTS1) at its C-terminus, which are PRL, SRL, SRL and SLL, respectively [30]–[31]. The accuracy of this prediction was experimentally tested by using a transient expression approach. The results showed that all of the GLO isoforms are localized to the peroxisome (Figure 7).

## Discussion

### Existence of GLO Isozymes and their Encoding Genes

While it is mostly considered that GLO exists as a single form consisting of an identical subunit (homo-oligomer) in plants [1], there have been reports showing that GLO isoforms exist in plants. As early as 1958, Frigerio and Harbury isolated 2 forms of GLO from spinach [18], one with a molecular weight of 140 kDa and the other 270 kDa. Havir (1983) noticed that the catalytic properties were changed during the GLO purification process [28], and considered the possibility that multiple enzymes are present for the oxidation of glycolate to glyoxylate. GLO isoforms have also been implicated in maize plants, where the isozymes were shown to be differentially distributed in bundle sheath and mesophyll cells [29]. In our current study, we not only firmly verifed the existence of GLO isozymes in rice, but also established that these isozymes are coordinately controlled by two genes, i.e., *GLO1* and *GLO4*.

Molecular aspects are still poorly understood for plant GLO, although cDNA sequences have been cloned from various plant species such as spinach, *Lens culinaris*, pumpkin, and *Arabidopsis* [32]–[35]. A search of the rice genome identified four putative *GLO* gene members, while five *GLO* genes were found in *Arabidopsis* (Table S1). It is of high value to understand how these genes contribute to GLO activities and its isozymes. Here we show lines of evidence that establish the existence of GLO isozymes and their encoding genes in rice. First, transcriptional analyses found that expressions of *GLO1* and *GLO4* predominate in rice leaves, while those of *GLO3* and *GLO5* predominate in the root (Figure 1B). When each gene was heterologously expressed in yeast, GLO1, GLO3 and GLO4 showed appreciable GLO activities but GLO5 was inactive (Figure 2). When the genes were pair by pair co-expressed in yeast, GLO1/GLO4, GLO1/GLO3 and GLO3/GLO4 showed almost additively increased activities, while co-expression of GLO5 with either of the others had no such effect (Figure 2). Further analyses demonstrated that GLO1, GLO3 and GLO4 interact with each other, while GLO5 does not (Figure 3, Table 1). Furthermore, the co-expression of GLO1/GLO4 in yeast was able to exhibit the same isozyme pattern as that seen in rice leaves (Figure 4). To further support this, an RNAi approach was applied to specifically silence *GLO1, GLO3* or *GLO4*. When either *GLO1* or *GLO4* was down-regulated, most of the GLO activities were lost, and both genes were simultaneously suppressed (Figure 5). In contrast, silencing of *GLO3* did not affect the GLO activities (Figure 5). All these results rule out the possibility that GLO3 is involved in GLO isozyme formation in rice.

Our results support the hypothesis that two *GLO* gene members, i.e., *GLO1* and *GLO4*, coordinately control the five GLO isozymes in rice leaves. There are a number of other enzymes which show similar behavior as GLO. For instance, two genes *LDH1* and *LDH2* encode five lactate dehydrogenase isozymes in barley [36]; and in *sorghum* endosperm, the two genes *SUS1* and *SUS2* contribute to five sucrose synthase isozymes through the random copolymerization of SUS1 and SUS2 subunits [37]. For glutamate dehydrogenase (GDH) in *Nicotiana plumbaginifolia* and *Arabidopsis*, seven GDH isozymes are encoded by two genes *GDHA* and *GDHB* [38]. It is also interestingly to note that *GLO1* and *GLO4* are coordinately suppressed at the mRNA level (Figure 5A), and similar results were also observed in *Arabidopsis* [9]. Such coordinated suppression also occurred for several other genes, including *RBCS* and *RBCL*, which encode the Rubsico small and large subunits, respectively [39]–[40].

While it has been long accepted that GLO is exclusively localized to peroxisomes, glycolate-oxidizing activities have also been detected in both the chloroplasts and mitochondria of plants [41]–[42]. Together with our current evidence for the existence of GLO isozymes and identification of different *GLO* genes in the rice genome, it will be interesting to reexamine the subcellular localization of GLO, with a particular regard to the various isozymes. Our results clearly demonstrate that all four of the GLO isoforms are localized to the peroxisome (Figure 7), and it can be logically inferred that the GLO isozymes in rice leaves should all localize to the peroxisome.

### Compositional Complexities for GLO Isozymes

To reveal the subunit composition of GLO isozymes, we originally tried preparing antibodies that would specifically recognize each isoform. However, because of the high similarity between the isoforms (Table S1), specific antibodies failed to be produced. In addition, purification of each individual isozyme was not successful, such that a mass spectrometry approach could not be applied to the identification of subunit composition. Alternatively, we used the BiFC and His-tag pull down assays to determine possible interactions between these isoforms. These analyses demonstrated that all possible interactions between GLO1, GLO3 and GLO4 occur, and that GLO5 did not interact with any of the other three GLOs (Figure 3, Table 1). This result suggests the possibility that GLO1, GLO3 and GLO4 constitute the entirety of the GLO isozymes. However, we also observed that *GLO3* abundance is very low in rice leaves, as compared to *GLO1* and *GLO4* abudance (Figure 1B). This obviously attenuates the possibility for GLO3 to be a subunit of GLO *in vivo*. By further comparing the GLO isozyme patterns in the rice leaves to the isoforms expressed in yeast, we found that the first and fifth isozyme are homo-GLO1 and homo-GLO4 polymers, respectively, and the other three isozymes are hetero-oligomers composed of GLO1 and GLO4.

The subunit size for GLO is reportedly similar in different plants, ranging from 38–43 kDa [20]–[21], [24], [32]. However, reports of the molecular weights of the holoenzymes differ greatly, ranging from 88 to 700 kDa, and even vary from study to study in the same plant species [18]–[27]. Some researchers attributed these variations to dissociation of the GLO holoenzyme during the purification process [20], [28]. In this study, we determined that the subunit size for all of the GLO isoforms is identical at 40 kDa (Figure 6A), and that the molecular weights of the holoenzymes range from 490 kDa to 650 kDa (Figure 6B). We thus deduced that the subunit number varies from 12 to 16, and that the first isozyme is a 12-mer composed uniformly of GLO1 and the fifth is a 16-mer composed uniformly of GLO4, and the second, third, and fourth isozymes are hetero-oligomers composed of GLO1 and GLO4, likely with only one subunit difference between them. In addition, we clearly observed that the GLO activities in rice leaves are mostly contributed by the hetero-oligomeric enzymes composed of GLO1 and GLO4 (Figure 1, 4). It has been commonly demonstrated that isozymes, particularly hetero-oligomeric ones, can play different biological roles. For instance, the fructokinase isozyme FRK1 functions in flowering in tomato, while the other isozyme FRK2 is involved in growth and development of different organs [43]. It is thus interesting to explore whether GLO isozymes may play additional unknown roles. While different isozyme patterns are seen in the *GLO4*-overexpressed lines (Figure 4), we have not yet seen changed isozyme profiles in response to environmental conditions (data not shown). Zelitch et al. (2009) found that *GLO* knockout mutants of maize (C_4_ plants) displayed the air-lethal phenotype similar to that seen in C_3_ plants, who holds the view that the photorespiratory pathway is equally important in C_4_ plants as it is in C_3_ plants [14]. Since photorespiration is known to be very minor in maize, another possibility still exists that GLO might play unknown essential roles, as has been previously proposed [17]. Further functional analyses are needed to explore the overall roles of GLO in rice and other plants.

## Materials and Methods

### Plant Materials

*Oryza sativa* cv. Zhonghua 11 (japonica cultivar-group) was used for constructing the transgenic lines and for the functional analyses.

### Growth Conditions and Treatments

Pre-germinated seeds were grown in Kimura B complete nutrient solution [44] or in normal soil under a greenhouse condition [average temperature of 30/25°C (day/night), relative humidity 60–80%, photosynthetically active radiation 600–1000 µmol m^−2^ s^−1^ and photoperiod of 14 h day/10 h night]. The second leaf from the top was detached and stored at −75°C for subsequent analyses.

### CN-PAGE and SDS-PAGE Analyses

To identify GLO isozymes and determine molecular weights of holoenzymes, a Caps-ammonium discontinuous CN-PAGE system with a running pH of 10.2 was used [45], which was developed from the commonly used Tris-gly discontinuous system with a running pH of 8.3 [46]. The uniform gel concentration is 6% and the gradient gel is 3–12%. Activity staining was carried out by incubating the gel at 30°C for 20 min in a staining solution which contained 0.016% (W/V) NBT, 0.003% (W/V) PMS, 0.1 mM FMN, 10 mM glycolate (pH 6–8), 100 mM phosphate buffer (pH 8.0). To determine the subunit size, purified proteins were fractionated on 12.5% SDS-PAGE gels and stained with CBB R-250.

### Heterologous Expressions of GLO Isoforms in Yeast

The complete ORFs of *GLO1*, *3*, *4* and *5* were cloned from rice leaves by RT-PCR and then respectively inserted into two vectors, i.e., pYES2/CT and pYES3/CT, where the latent tag was removed and 6 amino acids on the C-terminus of each gene was mutated to a 6-histidine tag for the purpose of subsequent purification. These constructed vectors were transformed into *Saccharomyces cerevisiae* INVSc1 (MATa *his3Δ1 leu2 trp1-289 ura3-52*) using the lithium acetate/carrier DNA method [47].

Positive clones were selected and transferred to 10 mL SC selective medium containing 2% glucose, and incubated at 30°C overnight with shaking at 250 rpm. Appropriate amount of overnight culture was then transferred to 50 mL SC inductive culture medium (SC selective medium containing 2% galactose) to obtain an OD600 of 0.4, and further incubated for 20 h under the same condition. The culture obtained was finally centrifuged at 4°C at 5000 rpm for 5 min, and the precipitate was collected and stored at −75°C for subsequent use.

### GLO Purification of and Determination of Its Catalytic Properties

The cultured yeast cells were suspended in a breaking buffer (50 mM sodium phosphate, pH 7.8, 5% glycerol) to obtain an OD600 of 50–100, and equal volume of acid-washed glass beads was added and the mixture was vortexed for 30 s and then placed on ice for 30 s. This step was repeated eight times to lyse the cells. Then the mixture was centrifuged for 10 min at 12000 rpm at 4°C, and the supernatant was collected and loaded onto a Profinity™ IMAC resin column (10 × 64 mm, from Bio-Rad). The column was first flushed at a flow rate of 1.0 mL/min with 10 column volumes of 50 mM PBS (pH 7.8) containing 300 mM NaCl and 10 mM imidazole, then the enzyme was eluted with 5 column volumes of 50 mM PBS (pH 7.8) containing 300 mM NaCl and 150 mM imidazole. The purification fractions were desalted by ultrafiltration and purity was checked by SDS-PAGE. Purification of GLO from rice leaves and determination of its catalytic properties were done according to Xu et al. (2006) [48].

### Interaction Assays

1. **BiFC assay.** The assay was performed mainly according to Bracha-Drori et al. (2004) with some minor modifications [49]. The ORFs of *GLO1*, *3*, *4*, *5* were inserted into the pSAT6-nEYFP-C1 vectors, while the p35s-cEYFP-*GLO* constructs were generated by fusing the ORFs of *GLO1*, *3*, *4* and *5* into the pSAT6-cEYFP-C1 vectors. NYFP-tagged constructs and CYFP-tagged constructs were co-transfected into the protoplasts with PEG. Then the fluorescent signals were detected by Olympus fluorescent microscope to determine whether the protein partners are fused.
2. **His-tag pull down assay.** Every two GLOs were co-expressed in yeast, and either was a his-tag fusion protein (Table S3). The protein-protein interaction complex was purified through immobilized metal affinity chromatography (IMAC), and then the recovery rate of glycolate oxidase activities was calculated to determine if the two gene products were interacted.

### Generation of *GLO*-Silencing and Overexpression Transgenic Lines

Primers were designed to amplify the interfering fragment to guarantee the specificity of the silencing (Table S2). Each specific fragment was then ligated into an RNAi vector named pYLRNAi.5. The vector is characterized by harboring two multi-cloning sites (MCS) so as to be more conveniently insert the target sequence in sense vs. antisense orientations. The cDNA fragment was firstly inserted in a sense orientation at MCS1 between *Sac*I and *Bam*HI. This first round ligated vector was then used as a template to amplify a second sequence with two unique restriction sites in ends. The second sequence was subsequently cloned at MCS2 between *Pst*I and *Mlu*I, thereby resulting in an opposite orientation in contrast to the sequence in MCS1. For overexpression of *GLO1*, *3*, *4*, *5*, the complete cDNA sequences for each gene were cloned by RT-PCR with the primer pairs as listed in Table S2, then the sequences were inserted into an overexpression vector named pYLox.5. First, PCR with specific primers and cutting with restriction enzymes showed that the target fragment had been correctly ligated. DNA sequencing finally confirmed the correct orientation and 100% cDNA identity to that reported in the GeneBank. The constructed interference and overexpression vectors were then transformed into rice callus, respectively, by *Agrobacterium*-mediated infection (strain EHA105). T_0_ lines were analyzed by Southern blot. T_1_ seeds from T_0_ lines with a single T-DNA insertion were grown to produce T_2_ seeds. Further screening with hygromycin-resistance gained the homozygous lines.

### Semi-Quantitative RT-PCR and Real-Time Quantitative PCR

Total RNA was isolated using TRIZOL reagent. One microgram of RNA was used as a template for first-strand cDNA synthesis using ReverTra Ace (Toyobo, Osaka, Japan) with random hexamers according to the manufacturer’s instructions. For semi-quantitative RT-PCR analysis, the optimal number of PCR cycles was first tested gene by gene. The PCR products were separated on 1% (w/v) agarose gels and visualized by Goldview staining. Quantification of PCR products were made by densitometry analysis using a computerized image analysis system, Quantity One (Bio-Rad, Hercules CA, USA). For real-time quantitative RT-PCR, specific primer pairs were designed using Primer Premier 5.0 (Premier Biosoft, Palo Alto, Canada). The PCR reaction consisted of 10 µL of 2×SYBR Green PCR Master Mix (Toyobo), 200 nM primers, and 2 µL of 1∶40-diluted template cDNA in a total volume of 20 µL. No template controls were set for each primer pair. The analysis was conducted by a DNA Engine Option 2 Real-Time PCR Detection system and Opticon Monitor software (Bio-Rad, USA).

### Quantification of Proteins

Protein concentrations of crude extracts were determined according to Bradford (1976) [50], the purified proteins were quantified by a NanoDrop ND-1000.

## Supporting Information

Table S1 **Similarity of GLOs in rice and** ***Arabidopsis thaliana.***
(DOC)

Table S2 **The primer sequences for amplifying the interfering fragments and the ORFs of** ***GLO*** **genes.**
(DOC)

Table S3 **The vectors constructed for the His-tag pull down assay.**
(DOC)
