# Supplementary material for: Glycolate Oxidase Isozymes Are Coordinately Controlled by GLO1 and GLO4 in Rice
Source: PLoS One. 2012 Jun 26;7(6):e39658. doi: 10.1371/journal.pone.0039658 (PMC3383670; doi:10.1371/journal.pone.0039658)
Supplement: Table S1 — Similarity of GLOs in rice and Arabidopsis thaliana. (DOC) [file pone.0039658.s001.doc]

**Table S1. Similarity of GLOs in rice and *Arabidopsis thaliana.***

| Protein | OsGLO1 | OsGLO3 | OsGLO4 | OsGLO5 |
| --- | --- | --- | --- | --- |
| AtGLO1 | 85.6% | 81.8% | 89.3% | 57.8% |
| AtGLO2 | 83.7% | 79.5% | 87.6% | 58.2% |
| AtGLO3 | 60.4% | 59.2% | 60.1% | 64.0% |
| AtGLO4 | 61.5% | 60.5% | 61.2% | 64.5% |
| AtGLO5 | 84.8% | 83.5% | 82.0% | 59.6% |
| OsGLO1 | 100.0% | 83.3% | 89.7% | 58.9% |
| OsGLO3 | 100.0% | 100.0% | 85.1% | 57.9% |
| OsGLO4 | 100.0% | 100.0% | 100.0% | 58.1% |
