# Supplementary material for: Glycolate Oxidase Isozymes Are Coordinately Controlled by GLO1 and GLO4 in Rice
Source: PLoS One. 2012 Jun 26;7(6):e39658. doi: 10.1371/journal.pone.0039658 (PMC3383670; doi:10.1371/journal.pone.0039658)
Supplement: Table S2 — The primer sequences for amplifying the interfering fragments and the ORFs of GLO genes. (DOC) [file pone.0039658.s002.doc]

**Table S2. The primer sequences for amplifying the interfering fragments**

**and the ORFs of *GLO*** genes.

| Gene | Primer sequences |
| --- | --- |
| Ri*GLO1* | 5’-CCTTGGATCCAGGCCGGGGTGAAGGAACGTGC-3  5’-AGTTAAGCTTTGAACGACCCAGTTACGAG-3’ |
| Ri*GLO3* | 5’-AGGAAGCTTAGGCTAAGCTCTTCAGCGGTTG-3  5’-CCCGGATCCCAAACTACTCTGAATCACCAAAT-3 |
| Ri*GLO4* | 5’-CTGGAAGCTTTAGAGCAGCAATGCACGTG-3’  5’-AACTGGATCCGAGTGAAGAGCCACGCAAG-3’ |
| Ri*GLO5* | 5’- TTAGAGCTCCGCCATAATGGTTCTTTCC -3’  5’- GTTAAGCTTAGCGGTGACGATgCCCTT -3’ |
| Ox*GLO1* | 5’-GAGAGAGGATCCATGGGGGAGATCACCAATGTCATG-3’  5’- AATTAAACGCGTCACAACCTGGGGAAGGGGCG-3’ |
| Ox*GLO3* | 5’ -TAAGGTACCCCTGAGACCGAAGAAAT-3’  5’ -CATAAGCTTGACAATTCGATCACTGC-3’ |
| Ox*GLO4* | 5’ - CCTGGTACCAGAGTGGTGTAGGTGATTCG -3’  5’ - TCTACGCGTGCAGCTACTACCTTCTTC -3’ |
| Ox*GLO5* | 5’ - GGCGGTACCCTTCCTCCCCTAAATCTCT -3’  5’ - ACAACGCGTAATAgATTCACGGGTCGGT -3’ |
