# Supplementary material for: Glycolate Oxidase Isozymes Are Coordinately Controlled by GLO1 and GLO4 in Rice
Source: PLoS One. 2012 Jun 26;7(6):e39658. doi: 10.1371/journal.pone.0039658 (PMC3383670; doi:10.1371/journal.pone.0039658)
Supplement: Table S3 — The vectors constructed for the His-tag pull down assay. (DOC) [file pone.0039658.s003.doc]

**Table S3. The vectors constructed for the His-tag pull down assay.**

| Co-expressed genes | Transformants |
| --- | --- |
| *GLO1*-his+*GLO3* | pYES2/*GLO1*-his+ pYES3/*GLO3*-INVSc1 |
| *GLO1*-his+*GLO4* | pYES2/*GLO1*-his+ pYES3/*GLO4*-INVSc1 |
| *GLO3*-his+*GLO4* | pYES2/*GLO3*-his+ pYES3/*GLO4*-INVSc1 |
| *GLO4*-his+*GLO1* | pYES2/*GLO4*-his+ pYES3/*GLO1*-INVSc1 |
| *GLO4*-his+*GLO3* | pYES2*/GLO4*-his+ pYES3/*GLO3*-INVSc1 |
| *GLO5*-his+*GLO1* | pYES2*/GLO5*-his+ pYES3/*GLO1*-INVSc1 |
| *GLO5*-his+*GLO3* | pYES2/*GLO5*-his+ pYES3/*GLO3*-INVSc1 |
| *GLO5*-his+*GLO4* | pYES2/*GLO5*-his+ pYES3/*GLO4*-INVSc1 |

*GLO*-his means the 6 amino acids on the C-terminus of GLO was mutated to a 6×his-tag. All of these transformants were incubated in the SC minimal medium that is deficient in tryptophan and uracil.
